# Validated Prognostic Nomograms for Patients With Parotid Carcinoma Predicting 2- and 5-Year Tumor Recurrence-Free Interval Probability

**DOI:** 10.3389/fonc.2020.01535

**Published:** 2020-08-25

**Authors:** Sam Peeperkorn, Jeroen Meulemans, Charlotte Van Lierde, Annouschka Laenen, Matthijs H. Valstar, A. J. M. Balm, Pierre Delaere, Vincent Vander Poorten

**Affiliations:** ^1^Otorhinolaryngology - Head and Neck Surgery, Leuven Cancer Institute, University Hospitals Leuven, Leuven, Belgium; ^2^Section Head and Neck Oncology, Department of Oncology, KU Leuven, Leuven, Belgium; ^3^KU Leuven Department of Public Health and Primary Care, Leuven Biostatistics and Statistical Bioinformatics Centre (L-BioStat), Leuven, Belgium; ^4^Head and Neck Surgery and Oncology, The Netherlands Cancer Institute, Amsterdam, Netherlands; ^5^Department of Oral and Maxillofacial Surgery, Amsterdam University Medical Center, Amsterdam, Netherlands

**Keywords:** oncological outcome, nomograms, parotid carcinoma, prognosis, tumor recurrence-free interval

## Abstract

**Introduction:** Salivary gland malignancies are rare tumors with a heterogenous histological and clinical appearance. Previously, we identified multiple prognostic factors in patients with parotid cancer and developed prognostic indices which have repeatedly been validated internationally, demonstrating their general applicability and lasting relevance. Recently, nomograms gained popularity as a prognostic tool. Thus, in this research we aimed to construct nomograms based on our previous validated prognostic models.

**Material and Methods:** Nomograms were constructed using the previously reported dataset of 168 patients with parotid cancer which was used to develop pre- and postoperative prognostic scores, PS1 and PS2, respectively. Concordance indices for PS1 and PS2 were previously estimated at 0.74 and 0.71, respectively, and are in line with other, widely accepted oncological nomograms.

**Results:** Pre- and postoperative nomograms predicting 2- and 5-year tumor recurrence-free survival probability are presented. All previously multivariately identified and validated prognostic factors, are incorporated (T size, N classification, pain, age at diagnosis, skin invasion, facial nerve dysfunction, perineural growth, and positive surgical margins). Examples of clinical application and interpretation are given.

**Conclusions:** The presented prognostic nomograms for predicting 2- and 5-year tumor recurrence-free probability in patients with parotid cancer are powerful, user-friendly, visual tools and are based on internationally validated prognostic indices. They allow for a reliable prognostic assessment and result in a more individualized estimate of the risk for recurrence than the prognostic grouping based on PS1 and PS2. This facilitates assigning trial-patients to risk groups, and may assist in therapeutic decision making and determining appropriate follow-up intervals in clinical practice.

## Introduction

Salivary gland malignancies (SGM) are a rare entity with an incidence which varies geographically between 0.05 and 1.9 per 100.000 per year (1). The incidence in the Netherlands in 2010 was 0.9 per 100.000 per year, with a tendency toward an increase over the last decades (2). In Belgium, the 2013 incidence was 0.7 per 100.000 per year (3). Staging of SGM is complicated by the vast anatomical area they can arise in and the heterogenous histological appearance (4). Nowadays, if the tumor and eventual regional metastasis are resectable, surgery is the standard of care (5, 6). Depending on the histopathological results adjuvant radiotherapy can be necessary. The clinically negative neck can be electively treated in high risk patients with radiotherapy or elective neck dissection (7). Chemotherapy is a treatment option reserved for palliative settings.

For parotid carcinoma, 10 year disease specific survival rates after treatment in major centers range from 47 to 69%, depending on population, study and disease characteristics (8). For the purpose of determining the expected treatment result of a specific patient within this range, many prognostic factors have been identified, and clinical prognostication tools have been developed (9–13). Increasing precision in estimating prognosis can aid in clinical decision making: it can help in deciding on treatment intensity and can help determining follow-up intervals. By informing patients on potential outcomes, shared-decision making is stimulated. Finally, prognostic information can help establishing different prognostic groups to use for stratification in observational studies and clinical trials (14).

To this purpose, we previously developed prognostic indices for patients with parotid cancer who receive surgical treatment, radiotherapy and/or chemotherapy (15). Clinical tumor size, N classification, pain, increasing age at diagnosis, presence of skin invasion, facial nerve dysfunction, perineural growth, and positive surgical margins were identified as independent negative prognostic factors. Based on these variables, pre-operative and post-operative prognostic scores (respectively, PS1 and PS2) predicting the tumor recurrence-free interval were developed. Using these scores, patients can be categorized into one of four subgroups with significantly different prognoses. Five-year tumor recurrence-free percentages ranged from 92 to 23% (in the group PS1 = 1 to PS1 = 4, respectively), and 95–42% (in the group PS2 = 1 to PS2 = 4, respectively). Subsequently, these prognostic indices were validated externally in a Dutch national database (NWHHT), in an international Belgian-German cohort, in an Asian cohort and most recently in an Italian cohort in 2018. These validations support general applicability and relevance as a prognostic tool in parotid cancer (11, 15–18). Until now, the prognostic indices were presented using a user-friendly web-based fill-out program with drop-downboxes, providing a calculated index PS1 and PS2 and the corresponding recurrence-free percentages and Kaplan-Meier curves (19).

Recently, the use of nomograms as a practical prognostic tool has become increasingly popular. Prognostic nomograms are visual representations of a complex mathematical formula or model, allowing for a more individualized risk assessment (20). For parotid carcinoma specifically, validated nomograms on tumor recurrence were not yet available. In this report, we present prognostic nomograms for estimating 2-and 5-year tumor recurrence-free interval probability before and after treatment, based on the data underlying our previously reported internationally validated prognostic scores (15).

## Patients and Methods

### Dataset and Prognostic Factors

The predictive nomograms were constructed from the original dataset that was used to develop the previously presented indices. This dataset included a retrospective cohort of 168 patients with a pathologically confirmed diagnosis of a malignant parotid tumor who visited the Netherlands Cancer Institute during the period from January 1, 1973 until December 31, 1994 (15). Of this group, 151 curatively treated patients were evaluated for tumor recurrence (local, regional and/or distant). In total, there were 79 women (47%) and 89 men (53%) with a median age of 63 years at diagnosis. Median follow-up time for patients alive at the end of follow-up was 94 months. Patients were treated according to a standard therapy protocol of the Netherlands Cancer Institute, consisting of surgery followed by radiotherapy. Patients with advanced disease, inoperability or other contraindications for surgery were treated with radiotherapy and/or chemotherapy.

Previously, clinical T classification, clinical N classification, pain, age at diagnosis, skin invasion, facial nerve dysfunction, perineural growth, and positive surgical margins were identified as prognostic factors for tumor recurrence using a multivariate proportional hazards analysis (15). As they emerged as significant in the multivariate analysis and thus were included in the model, each factor was assigned a weight based on the regression coefficients, associated with risk of tumor recurrence. These clinicopathological factors and their respective weights were then combined in one mathematical formula from which the pre- and post-operative prognostic index were derived (PS1 and PS2, respectively). The prognostic indices were then validated by our own research group in two separate, external validation cohorts of patients with parotid carcinoma (16, 17). Predictive accuracy was assessed using the concordance-index (CI), which is a measure of predictive accuracy and represents the proportion of pairs of patients in whom highest prognostic score indeed implies shortest disease free interval. Firstly, this was done in the Dutch Head and Neck Cancer Cooperative Group (NWHHT) database, which contained 231 consecutive patients from six tertiary referral centers in the Netherlands, resulting in a CI of 0.71 and 0.74 for PS1 and PS2, respectively (16). Secondly, the prognostic indices were validated in a Belgian-German database that contained 237 consecutive patients, resulting in a CI of 0.74 for both PS1 and PS2 (17). Later on, the prognostic indices have also been validated externally by independent international research groups. A Taiwanese research group found a CI of 0.74 for both PS1 and PS2, and most recently an Italian group published a CI of 0.73 for PS1 and 0.79 for PS2 (11, 18).

In the previously reported prognostic indices, tumor size and regional lymphatic spread was described using T- and N-classification, respectively, from the UICC TNM 4th edition (21). Since the 4th edition there have been major alterations in both T- and N- classification level definitions. As T-classification has completely changed in current editions, we kept in our nomograms the respective tumor size categories (<2, 2–4, 4–6, >6 cm) that defined T-status in the 4th edition. Regarding N-classification, as extra-nodal extension was added in the 8th UICC TNM edition and a new category, N3b, was created, it could not be used as such. For this reason, we use the N-status definitions of the still widely used UICC TNM 7th edition, as N-classification level definition remained unchanged from the 4th through the 7th edition (22).

### Nomogram Construction

Prognostic nomograms for 2- and 5-year tumor recurrence-free probability in a pre- and post-operative setting were constructed using the rms-package in RStudio software (RStudio, Boston, USA), a graphical user-interface for R programming language (R Foundation for Statistical Computing, Vienna, Austria (23). Nomograms were created based on a Cox proportional hazards model associated with a formula of the following form:

$$\begin{array}{r} \text{Probability of tumor recurrence at time }t\;=\;{\text{R}}_{0}{\left(t\right)}^{\text{exp}\left(\text{β}1\text{x}1+\text{β}2\text{x}2...\right)}. \end{array}$$

where R_0_(*t*) is the baseline hazard function for tumor recurrence, corresponding to the risk of tumor recurrence when all covariates are zero, which was estimated from the original dataset (20). Two nomograms were constructed building upon the models underlying PS1 and PS2, i.e., retaining their prognostic factors (x_1_, x_2_,.) and their corresponding regression coefficients (ß_1_, ß_2_,.). The regression coefficients, which equal the natural logarithm of the hazard ratios, were used to construct the variable axes in the nomogram with each value correlating to a number of points from 0 to 100. Finally, total points accounting for cumulative hazard were translated to predicted tumor recurrence probability using the baseline hazard function (24).

## Results

**Figure 1:**
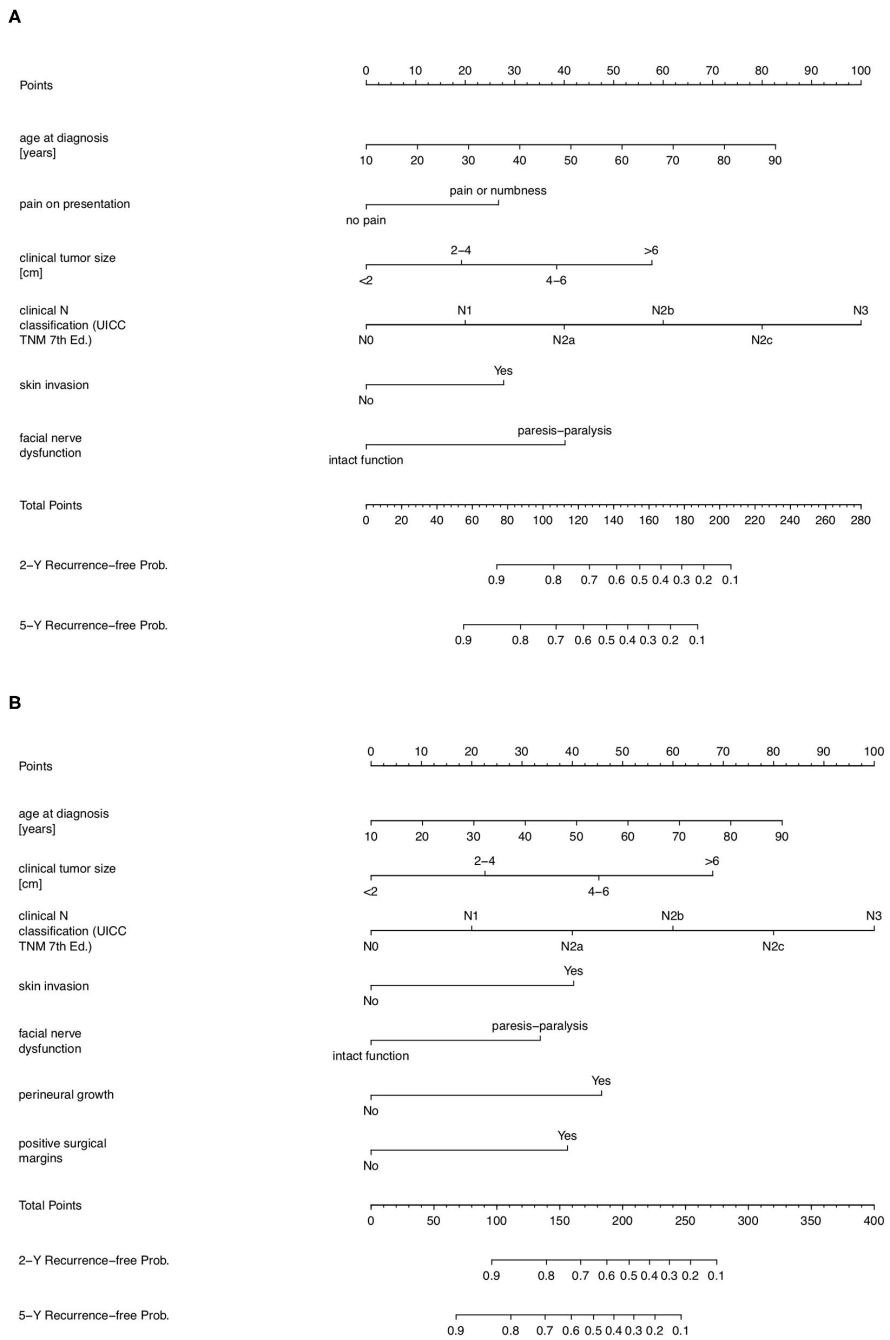
Prognostic nomograms for predicting 2- and 5-year tumor-recurrence free interval probability after treatment in patients with parotid cancer. **(A)** Prognostic nomogram in a pre-operative setting, constructed from the PS1-score. **(B)** Prognostic nomogram in a post-operative setting, constructed from the PS2-score.

**Figure 2:**
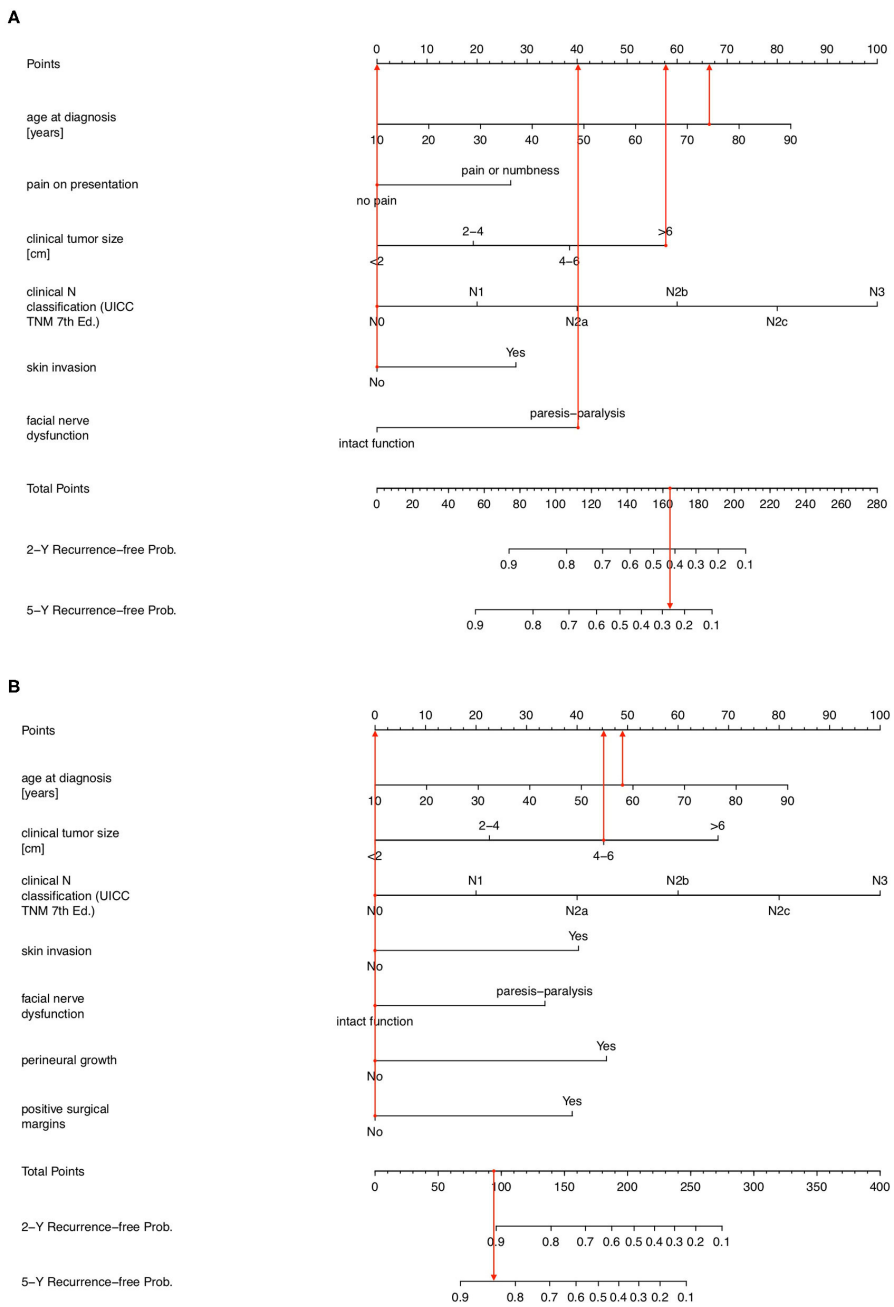
**(A)** Prognostic nomogram showing pre-operative probability of being 2- and 5-year tumor-recurrence free in a 74-year female with a painless 6.5 cm cT4aN0M0 adenoid cystic carcinoma with progressive facial nerve palsy but without skin invasion. **(B)** Prognostic nomogram showing post-operative probability of being 2- and 5-year tumor-recurrence free in a 58-year male with a 46 mm painless muco-epidermoid carcinoma without facial nerve palsy or skin invasion with negative resection margins, no perineural growth and pT3N0M0 on the final histological report.

Nomograms predicting 2- and 5-year tumor recurrence-free interval, incorporating all previously identified prognostic factors were constructed, based on both pre-operative (PS1) and post-operative (PS2) prognostic scores; (Figure 1). By drawing lines on the nomogram, the corresponding points can be identified and added to obtain a total score. This score then corresponds to an individual estimate of a 2- or 5- years recurrence free percentage. As an example, Figure 2A shows the nomogram used for a 74-year old female patient with a painless 65 mm (cT4a), cN0, cM0 parotid adenoid cystic carcinoma with progressive facial nerve palsy, without skin invasion. Drawing the lines, a pre-operative score of around 164 points correlates with a 42 and 27% probability of being recurrence-free at 2- and 5-years follow-up, respectively. This patient received a maximally radical treatment, consisting of total parotidectomy and neck dissection of levels I-III and a full course of adjuvant radiotherapy (66 Gy).

Example 2 considers a 58-year old male patient with a 52 mm (cT3), cN0, cM0 low grade muco-epidermoid carcinoma with no pain, no facial nerve dysfunction nor skin invasion, which on the postoperative pathology report shows no perineural invasion and negative surgical margins (Figure 2B). Using the nomogram, the total score is 93 points, corresponding to a 2- and 5-year tumor recurrence-free probability of 91 and 84%, respectively, when treated with standard of care. This patient received adjuvant radiotherapy as well, given the advanced local extension at diagnosis.

Considering example 1, according to the previously developed PS1 score, the patient would be classified in group 4 with a 22.8% (9% SE; confidence interval 8.5–41.2%) 5-year tumor recurrence-free survival probability. The predictive nomogram now provides a more individualized and higher pre-operative prognostic estimate of a 27% chance of being recurrence-free at 5-year follow-up, which still lies within the 95% confidence interval. In example 2, the patient would be classified prognostic group PS2 = 2, corresponding to an 82.5% (SE 8%; confidence interval 59.8–93.1%) chance of being tumor free at 5 years. The 5-year tumor recurrence-free interval probability according to the postoperative prognostic nomogram is in line with this but indicates an individualized 84% chance of being cured at 5 years after standard of care treatment, in this case surgery and adjuvant radiotherapy. It provides a somewhat more specific answer to the patient's question of how likely the performed treatment is to cure the disease.

## Discussion

Currently, the most widely-used prognostic tool for all solid tumors, including SGM, is the TNM staging system (25). This system categorizes patients into different stages based on anatomical disease extent, without inclusion of other important patient- or disease-related variables such as age, pain, perineural invasion, or tumor grade (15). Previously, we developed prognostic scores for patients with parotid cancer which do incorporate these other patient- and disease-related factors (15). These scores were subsequently validated in external datasets. In a dataset from the Dutch Head and Neck Cancer Cooperative Group (NWHHT), the resulting CI for PS1 and PS2 were 0.74 and 0.71, respectively (16); in a Belgian-German database, CI were 0.74 for both PS1 and PS2 (17). These CI are in line with other, widely accepted nomograms of other cancers (20). Furthermore, when repeatedly validated internationally, the performance of the prognostic scores remained good, with the latest CI being 0.74 for PS1 and 0.79 for PS2, supporting the lasting clinical relevance and general applicability of PS1 and PS2 (10, 17).

In line with the current gaining popularity of nomograms and based on the multivariate analysis that resulted in the validated prognostic indices, we now present user-friendly nomograms for estimating 2- and 5-year tumor recurrence-free interval probability after standard of care treatment of patients with parotid cancer.

Recently, three other interesting prognostic nomograms regarding tumor recurrence following SGM treatment have been developed, all from a single center cohort.

Authors from the Memorial Sloan Kettering Cancer Center (MSKCC) presented nomograms predicting 5 year recurrence probability after treatment for all major SGM, based on a multivariate analysis including age, grade, vascular and perineural invasion, and nodal metastasis (26). Validation on a separate cohort from the same institution showed a very good CI of 0.84 (27). Performance in a true external validation in Taiwan showed a very good CI of 0.82 too, although for advanced stage tumors (T3-T4) the CI was less impressive (28).

The Taiwanese group also developed their own nomogram for salivary gland tumor recurrence based on tumor size, nodal involvement, perineural invasion, tumor grade, lymphatic invasion and smoking status (29). Validation of the nomograms was performed on a different cohort from three affiliated hospitals in the region and showed a CI of 0.78 for 2- and 5-year tumor recurrence probability. However, as indicated by the authors, the validation was performed using a national database so international external validation is still desirable.

More recently, Mannelli et al. presented a postoperative prognostic nomogram for 5 year parotid cancer recurrence probability, with excellent CI as well (0.829; as compared to our initial PS2 CI of 0.79) (13). Variables used to construct this nomogram include age, stage, histological grade, perineural invasion and pathological lymph node status. However, this predictive nomogram has not yet been validated externally.

In comparison, our nomograms specifically focus on the entire group of parotid gland malignancies and are based on prognostic indices that have been validated internationally (11, 16–18). We additionally provide a pre-operative nomogram which could be of value in counseling patients before deciding on the treatment. Finally, our prognostic nomograms include pain, skin invasion, facial nerve dysfunction and positive surgical margins as additional prognostic factors to explain the observed variability. Histological grade was not included as it lost its significance in the original predictive model when the age factor was introduced (15). This could be explained by the positive association between the two (chi-square for trend *P* < 0.001), which is also described in recent literature (30).

Nomograms have a few intrinsic limitations. First, they risk giving a false feeling of individualized and precisely estimated prognosis. Indeed, most of the underlying prognostic factors to build a nomogram are categorical and consequently create patient subsets which compromise later precision in individualization using the summary information of a nomogram. The previously reported prognostic scores PS1 and PS2 are statistics “on group level,” yielding 5 year recurrence-free probability estimates with broad confidence intervals, reflecting maybe more the caution one has to take in interpreting these estimates. This is in contrast with the individual outcome without confidence interval that the predictive nomogram suggests. In line with this and as pointed out above, a difference in probability between the prognostic indices and corresponding nomograms was observed. Second, in order to correctly use nomograms for clinical decision making and counseling of patients, the user needs to fully understand and be able to interpret nomogram performance (e.g., discrimination) and limitations. Also, the effects of nomogram-assisted decisions on patient satisfaction and clinical outcome still remain unclear (20).

One last limitation of the currently presented nomograms is that the initial multivariate model was based on the 1992 TNM classification (31). Over the years there have been alterations where the presence of invasion of soft tissue surrounding the salivary gland or affected lymph node result in a higher T- and N-category, respectively (25). This limitation is addressed by using the tumor size categories (<2, 2–4, 4–6, >6cm) and the UICC TNM 7th edition node classification for N status, as already clarified in the Patients and Method section.

In the future, research incorporating other possibly important prognostic variables such as patient comorbidity, histological subtypes and underlying genetic mechanisms (as reflected in molecular biological markers) can improve prognostication models (32). Performing this research based on multi-center cohorts rather than single-center cohorts could also increase international applicability and clinical usefulness of prognostic tools. To further enhance clinical user-friendliness, the prognostic indices and nomograms could be incorporated in a computer or smartphone application, allowing for instant prognostic score calculation.

In conclusion, the presented prognostic nomograms provide a scientifically based and externally validated prediction of 2- and 5-year recurrence-free interval after standard of care treatment in patients with parotid cancer, in a pre- or postoperative setting. They are user-friendly visual representations of complex prognostic models to aid in individual prognostication, clinical decision making, and may serve as a basis for risk stratification in clinical trials.

## Data Availability Statement

The data analyzed in this study is subject to the following licenses/restrictions: The data upon which the research is performed are available upon reasonable request to the corresponding author provided that (1) an EC approved research protocol can be presented and (2) all collaborating centers agree to share the data. Requests to access these datasets should be directed to Vincent Vander Poorten, vincent.vanderpoorten@uzleuven.be.

## Author Contributions

VV was responsible for the initial concept of this paper. SP wrote the manuscript and made the figure outlines with the aid of VV. AL created the prognostic nomograms and provided feedback and suggestions from a statistical point of view. All other authors provided valuable feedback, suggestions, and corrections to improve the quality of the manuscript.

## Conflict of Interest

The authors declare that the research was conducted in the absence of any commercial or financial relationships that could be construed as a potential conflict of interest.

## Abbreviations

SGM: salivary gland malignancies
PS1: pre-operative prognostic score
PS2: post-operative prognostic score
CI: concordance-index.
